# Nutritional vulnerability: An assessment of the 2010 feeding food programme in Mbire district, Zimbabwe, and its impact on pregnant women

**DOI:** 10.4102/jamba.v9i1.406

**Published:** 2017-08-11

**Authors:** Alice Ncube, Olivia Kunguma, Moddie Nyahwo, Stella Manombe

**Affiliations:** 1 Disaster Risk Management Training and Education Centre for Africa, University of the Free State, South Africa

## Abstract

Malnutrition contributes significantly to Zimbabwe’s high maternal mortality rate. The prevalence of malnutrition among vulnerable pregnant women in the Mbire district of Zimbabwe was studied to establish why they remained vulnerable despite benefiting from the Vulnerable Group Feeding Programme, a subsidiary of the World Food Programme. A case study on the demographic characteristics, nutritional provision of the programme and the vulnerable pregnant women benefiting from the programme was conducted. One hundred women were purposively sampled at health centres in the district. A two-stage sampling procedure was then utilised to select the most food-insecure wards. The two most food-insecure wards, namely Angwa and Chapoto, were chosen because of their proximity to each other. A questionnaire was administered to the pregnant women to collect their demographic information. Practising nurses at the health centres determined the women’s nutritional status and anthropometrics, and they also assessed the food baskets. Permission to conduct the study was obtained from the relevant authorities. The results indicated that the food hamper provided by the World Food Programme was complementary food aid given to all vulnerable members of the community regardless of the nutritional demands. The supplements that the pregnant women received were also inadequate to cater for their nutritional needs or those of the foetuses. It was therefore recommended that the government, through the Ministry of Health, should make more provisions available for vulnerable pregnant women in order to reduce the risks facing pregnant women in the country.

## Introduction

According to Napoli (2011) Zimbabwe’s commercial maize (staple food) planting was drastically reduced from 150 000 ha to 45 000 ha during the 1999–2000 agricultural season. This was partly because of the Fast Track Land Reform Programme implemented by the Zimbabwean government between 2000 and 2002 (Moyo 2010). Little maize was produced by the communal farmers, who depended mainly on employment and supplementary maize from commercial farmers. Richardson (2004) confirmed this by stating that agricultural yields in Zimbabwe had fallen considerably since 2000. Thus, the food security in Zimbabwe was becoming increasingly compromised and the government had to import food from outside the country. In the 2005–2006 agricultural season Zimbabwe failed to produce enough cereals to meet the country’s needs (USDA Foreign Agricultural Services 2005). Because of the political and mostly economic challenges the country was facing, it became impossible for Zimbabwe to import adequate amounts of cereals to cover the deficit. The World Food Programme (WFP) initiated the Vulnerable Group Feeding (VGF) programme, which was designed to cushion more than 3.4 million food-insecure Zimbabweans (WFP 2005). Since then, the programme has been implemented every year because of recurring droughts and food insecurity facing southern Africa and Zimbabwe in particular. By 2009 Zimbabwe’s food situation had deteriorated more drastically than expected, because of the worsening cash crisis and political situation resulting in many people needing food aid. Mutisi (2009) further confirmed that since 1995 food production in Zimbabwe had declined steadily, resulting in food importation and upsurge in food aid needs. The WFP intervened and intensified the distribution of food to vulnerable communities in Zimbabwe. The VGF programme, a programme aimed at alleviating short-term hunger (Médecins Sans Frontières 2006), was rolled out to many places throughout the country, which were considered to be food insecure. One such food-insecure area was the Mbire district, which benefited from the programme. The purpose of the study was to assess how the VGF programme impacted on the nutritional vulnerability of the pregnant women in Mbire district, Zimbabwe. Table 1 shows the commodity list of the VGF programme basket intended to relieve short-term hunger (WFP 2000).

**TABLE 1:** Vulnerable Group Feeding Programme food basket.

Commodity	Energy (kcal)	Protein (g)	Fat (g)
**Cereals**			
Rice	360	7.0	0.5
Sorghum/millet	335	11.0	3.0
Maize	350	10.0	4.0
**Processed cereals**			
Maize meal	360	9.0	3.5
Bulgur wheat	350	11.0	1.5
**Oils and fats**			
Vegetable oil	885	-	100.0
**Pulses**			
Beans	335	20.0	1.2
Peas	335	22.0	1.4
Lentils	340	20.0	0.6

*Source*: World Food Programme (WFP), 2000, *Food and nutrition handbook*, World Food Programme, Rome, Italy

According to a report by the International Food Policy Research Institute (2016), 2 billion people suffer from micronutrient malnutrition, while approximately 800 million do not receive sufficient calories in their diet. The report also records a stunting prevalence of 27.6% as well as a 28.4% anaemia prevalence in woman of reproductive age in Zimbabwe. The survey on the national nutritional status conducted in 2010 revealed that the prevalence of malnourishment in the country stood at 33.8% (Zimbabwe National Nutritional Survey 2010). According to the Zimbabwe Multiple Indicators Monitoring Survey (MIMS) report (2009) an infant death rate of between 8.7% and 13.7% was caused by undernourishment and HIV and/or AIDS, one of the highest in the world. Inadequate food and some diseases were immediate contributing factors of malnutrition, while socio-economic, political and water and sanitation were among some of the root causes. These were according to the United Nations Children Fund (UNICEF) (1997) framework for the underlying causes of malnutrition and mortality and were also confirmed by the WFP on nutrition policy in 2000, which emphasised that poor hygiene and unsuitable water can result in the outbreak of diarrhoeal diseases and worm infestation, causing malnutrition. Undernourished individuals can easily succumb to these vulnerabilities because of their weakened immune system (Médecins Sans Frontières 2006). Children and non-pregnant women were, however, included in these studies and thus the results reflect children’s health. As food insecurity results in malnutrition especially among the poor and vulnerable groups such as pregnant women, this study assessed the impact of the VGF programme on pregnant women in Mbire district in Zimbabwe.

## Malnutrition vulnerability among pregnant women

Malnutrition is the condition that develops when the body does not receive the right amount of the vitamins, minerals and other nutrients it needs to maintain healthy tissues and organ function (Sheetal et al. 2013). The existence of malnutrition and other related threats results in communities and individuals being nutritionally vulnerable (Hewitt et al. 2006). Nutritional vulnerability refers to a reduced physical reserve that limits recovery in the event of an acute health threat, as well as limited resilience and other predisposed factors arising from a reduced dietary intake (Starr, McDonald & Bales 2015). In many developing countries the effects of malnutrition on pregnant women are under-investigated because the focus is mostly on infant mortality and morbidity issues while neglecting the direct link between antenatal care and infant well-being (Hardee, Gay & Blanc 2012).

A study that Tomkins conducted in Nepal in 2001 concluded that maternal mortality rates can be drastically reduced by simply using vitamin B and A supplements. In Nepal also, it was established that there was a 44% reduction in maternal mortality because of this vitamin supplementation programme (Tomkins 2001). According to Insel and Wardlaw (1999), expectant mothers are more at risk if their diets do not supply the required nutrients. These nutrients are for the mothers’ nutrient needs and consequently the foetuses’ (Almurshed et al. 2007). It is therefore necessary that expectant women receive extra nutrients to meet their increased physiological needs. Pregnant women need extra kilocalories, iron and folates, which are scarce in situations where there is food insecurity (Ladipo 2000).

The majority of the malnourished groups are pregnant women living in developing countries in Africa (Democratic Republic of Congo, Ethiopia and Southern Sudan) and Asia (Bangladesh, China, India, Indonesia, Pakistan and China) (State of Food Insecurity [SOFI] Report 2010). China and India together account for 40% of the total malnourished groups that include pregnant women, which is contrary to the popular view that malnourishment is increasing only in Africa (Webb et al. 2006). Webb et al. made an estimate that 1 in 16 African pregnant women living in undernourished, unhealthy and inadequate maternal care environments are likely to succumb to pregnancy-related complications. Wisner et al. (2004) pointed out that during the 2002 drought in Malawi the WFP revealed that almost 70% of Malawians suffered from nutritional deficiencies. This contributed to a compromised immune system that made them more vulnerable to HIV and/or AIDS, cholera and bubonic plague, among other diseases. Pregnant Malawian women were particularly at risk, because of their malnutrition levels (Malawi Multiple Indicator Cluster Survey 2006). That brought to the fore the vulnerability of child-bearing women in the pre- and postnatal periods in some developing countries.

## The study area

**FIGURE 1:**
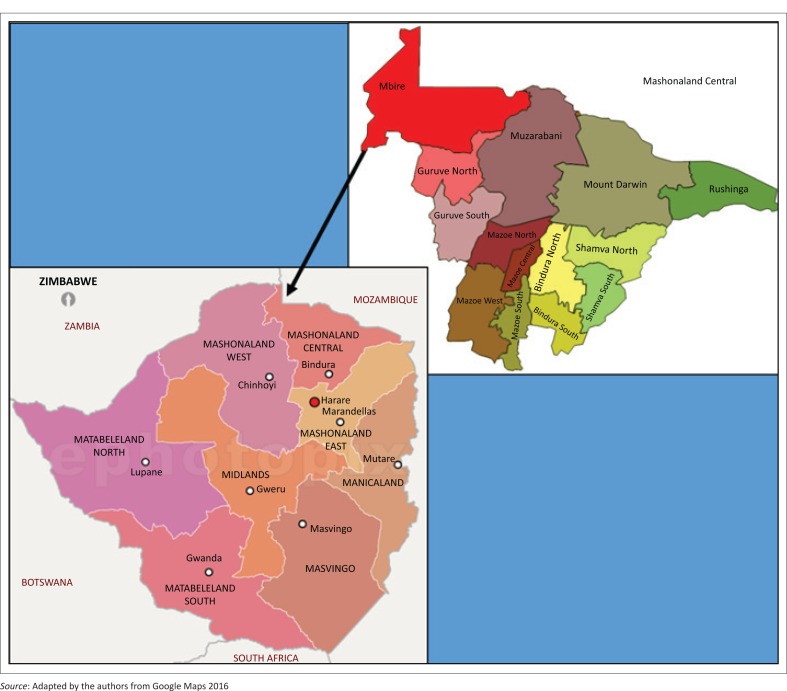
Map indicating the location of the Mbire district.

The Mbire district (Figure 1) was chosen as the study area because it is one of the four districts in Zimbabwe with 39% – 42% food-insecure households and hence part of the WFP-VGF programme (Zimbabwe Vulnerability Assessment Committee [ZIMVAC] 2010). The same report showed that most studies conducted in the Mbire district concentrated on food security, and no special attention was paid to the more vulnerable groups such as pregnant women. The district is divided into 17 wards. Of these, three wards, namely Chapoto (Ward 1), Angwa (Ward 2) and Masoka (Ward 11), were identified as the most food-insecure wards (ZIMVAC 2010). In that district the majority of the population depended on food aid because their agricultural livelihood practices were compromised by recurrent drought characterised by low, erratic rainfall with the average yearly precipitation ranging from 450 mm to 650 mm (Fritz et al. 2003). The peak rains are experienced in January and February, usually coinciding with the flooding of the rivers and resulting in poor crop yields. The area is characterised by highly unreliable rains with severe dry spells, recurrent seasonal droughts and high temperatures averaging 40 degree Celsius in summer (Food and Agriculture Organisation of the United Nations 2006). Additionally, the people of Mbire grow maize, which is not drought tolerant (Le Bel, Stromberg & Duckworth 2004).

## Research methodology

The case study was descriptive in nature, and both qualitative and quantitative data were collected. The total population of the Mbire district is about 115 000 with the people residing in 17 wards (Mbire district Baseline Survey Report 2009). Purposive sampling was used to select the 100 respondents for the survey, and the anthropometric assessments were conducted at the respective health centres. A two-stage sampling procedure was followed. Firstly, simple purposive sampling was used for the wards to minimise sampling error and bias (Saunders, Lewis & Thornhill 2003). The two most food-insecure wards were chosen, namely Angwa (1) and Chapoto (2). These were sampled because of their proximity to each other. The actual number of pregnant women at any given time was difficult to establish, and so the researchers relied on the information provided by the health department in the area. One hundred pregnant women who were attending monthly antenatal care at the health centres were also purposively selected to participate in the study. The reason for choosing 100 women was that, according to the nurse-in-charge, the population of the pregnant women in the area was homogenous as they were all food insecure and depended on food aid owing to the recurrent drought in the Mbire district. This choice is supported by Maree et al. (2007), who stated that lesser samples may sufficiently represent the population in homogenous populations, where participants are similar with respect to the variables that are central to the study.

The data were collected in the month of August 2011, a peak period when food shortages are experienced in the Mbire district. August was also ideal as it was a dry month and the people were able to travel to the health centres. In this way more women could be involved in the study. The rivers (Angwa, Manyame and Hunyani) and the bridges are usually inundated during the rainy season and people are confined to their homes (Madamombe 2004). The transport system in the area is also very poor with the gravel roads getting flooded and the bridges submerged. Before data collection commenced, the researchers obtained a letter of introduction from the University of the Free State, Disaster Management Training and Education Centre for Africa (UFS-DiMTEC). Permission to collect data was sought from the district Ministries of Local Government and Health and Child Welfare. The headman, representing traditional leadership structures in the area, was also approached to seek his permission as the respondents were his subjects. Respondents agreed to participate in the study and that their medical history could be used because no personal identities were required from them.

A semi-structured questionnaire was used to collect primary data consisting of demographics (age, level of education, marital status, religion, number of wives in the marriage and language of communication), maternal health (age of first pregnancy, total number of prior pregnancies, birth intervals, receipt of different supplements and distance to the clinic), water and sanitation issues, food basket composition, utilisation and satisfaction, assets and other livelihoods and finally anthropometric information (total meals per day, sickness in the past 14 days, Body Mass Index [BMI] and mid-upper arm circumference [MUAC]). Anthropometric measurements were carried out by one researcher who is a nurse and supported by nurses working in those clinics.

Data were collected over 2 days, during the period when the women from various settlements visited the clinics for their monthly antenatal check-ups. Face-to-face interviews using a semi-structured questionnaire were conducted with the pregnant women. The face-to-face interaction with the respondents was beneficial as it gave the researchers an opportunity to clarify issues that might be misconstrued and perhaps vague to some respondents. The face-to-face interaction also gave the researchers, via the researcher who was a state-registered nurse, the opportunity to do clinical assessments for clear signs of micronutrient deficiencies. The personal information identification of the respondents was not captured in the study. Observations and content analysis of the food basket were conducted as discussed further in the article. The nutritional content of the food rations distributed and nutritional risks associated with malnutrition in pregnant women were assessed. In addition to the observations, some of the anthropometric data of the pregnant women were obtained from the health centres.

After all the data had been collected the researchers checked for completeness of the questionnaires and verified if all the measurements were conducted correctly. After verification, the complete questionnaires were assigned identity codes and the quantitative data were then captured into a Microsoft Excel sheet and input into the Statistical Package for the Social Sciences (SPSS) Version 17. Summaries of the observations and qualitative data were entered into a word document and also arranged according to themes. The nutritional status of the respondents and the prevalence rates of malnutrition were estimated utilising the anthropometric data. The data were then interpreted, analysed and discussed in thematic forms and others were presented in tables, figures and graphic formats.

## Empirical findings

All of the 100 respondents were beneficiaries of the WFP-VGF programme. The demographic information of the respondents is given in Table 2. Most (34%) of the respondents were adolescents and youths (between 15 and 20 years), followed by 27% respondents who were between 21 and 30 years, 24% between 31 and 40 years, 3% under 15 years and 12% were above 40 years of age. The respondents in the 15–20 years age group and under 15 years were adolescent and young girls. According to Wardlaw (2000), pregnant adolescent women or young girls are at risk of exposure to maternal anaemia and pregnancy complications among many other nutritional deficiency-related difficulties. A study by Kazaura, Kidanto and Massawe (2006) found that adolescent and youth pregnancies were one of the causes of the high mortality rates among pregnant mothers in Tanzania. Wardlaw (2000) further states that this exposure is attributed to the women’s underdeveloped physical structures. Insel and Wardlaw (1999) then argue that the nutritional demands of an expecting woman who is also still developing require them to obtain sufficient nutrition. The young maternal age of some of the women in Mbire contributed to their nutritional vulnerability as confirmed by Insel and Wardlaw (1999), Wardlaw (2000) and Kazaura et al. (2006).

**TABLE 2:** Demographic information of pregnant women in the Mbire district.

Demographic information	Category	Percentage
Ages (years)	< 15	3
	15–20	34
	21–30	27
	31–40	24
	< 40	12
Highest level of education	Secondary education	20
	Primary education	52
	No schooling	28
Marital status	Married	71
	Divorced	4
	Widowed	10
	Single	8
	Other	7
Duration of stay in Mbire (years)	< 3	2
	3–5	17
	6–15	14
	> 15	67
Religious characteristics	Apostolic faith	28
	Protestant Christianity	21
	Traditional religion	43
	Other	8
Types of marriages	Polygamous	57
	Monogamous	43
Number of pregnancies before the current one	None	30
	1	16
	2	7
	3	14
	> 4	33

*n* = 100.

Other challenges, like previous pregnancies, might have an impact on the women’s nutritional fitness. A third (33%) of the respondents indicated that they had had more than four pregnancies before the current one. Thirty per cent of the respondents indicated that it was their first pregnancy. Others, 16%, had one child, 7% had two children and 14% had three children. The number of children that these women have has an effect on their nutritional vulnerability because of physical body deterioration. Short inter-pregnancy intervals cause depletion of nutrients, resulting in the mother and the unborn baby being malnourished (King 2003). In the USA it was found that women with inter-pregnancy intervals of less than 8 months were between 14% and 47% more likely to have very premature and moderately premature infants than women with intervals of between 18 and 59 months (Fuentes-Afflick & Hessol 2000).

Seventy-four per cent of the respondents indicated that they had had their first pregnancy when they had been between 15 and 17 years old. Respondents who first fell pregnant between 18 and 24 years were only 24%. Two per cent of the respondents had their first pregnancies between 21 and 25 years of age. Fuentes-Afflick and Hessol (2000) stated that young or adolescent women are not biologically prepared for conception especially if they are within 2 years of their menarche. Scholl et al. (1994) confirmed this when they found that young pregnant women competed for nutrients with their foetuses as they themselves would also still be growing during gestation.

According to Lilungulu et al. (2015), short inter-pregnancy intervals impact negatively on pregnant women’s nutritional health status. Nutritional risks, such as immunodeficiency and anaemia, are usually experienced by women who have inter-pregnancy intervals of less than one and a half years (Wardlaw 2000). This is because their nutrient stores have been diminished during the previous pregnancy and are not able to provide for the current pregnancy. Positively, this study discovered that a small number of the women, about 29%, experienced inter-pregnancy intervals of less than 2 years, while the majority of about 71% fell within the inter-pregnancy interval of more than 2 years. The poor health of the women cannot only be attributed to the shorter intervals between births. Other factors such as an inadequate diet could have contributed to this. Nevertheless the 71% women with longer inter-pregnancy intervals could also be nutritionally vulnerable.

As in many other African countries, as stated by Von Struensee (2005), the women from the Mbire district were not exempt from the challenges that come with polygamous marriages. Polygamy is part of the culture of many African communities (Anderson 2000). About 57 of the 100 respondents in the study indicated that they were in a polygamous marriage. In most polygamous marriages, the husband moves out from the pregnant woman’s house to live with the other wife or wives (Bove, Vala-Haynes & Valeggia 2014), hence the pregnant women’s increased nutritional vulnerability.

## Supplementary nutrients

The study ascertained whether the respondents received adequate nutrients in the form of vitamins, iron and folic acid, as these are critical to a pregnant woman for the growth of the unborn baby. According to the Zimbabwe National Nutrition Survey (2010), vitamin A supplementation coverage was 25.2% (189 μg retinol activity equivalents [RAE]), which was far below the internationally acceptable threshold of a minimum of 750 μg RAE (World Health Organization [WHO] 1995). Iron and folic acid were also far below the expected threshold of 30 mg – 60 mg of elemental iron and 0.4 mg of folic acid per day (WHO 2012). The results from the study could be insignificant because the mentioned low literacy levels indicated in Table 2 could have led to the miscomprehension of the question, when the women actually received the supplements without knowing them. To help triangulate these results, the researcher had to investigate this issue further. It was confirmed by the nurses that supplementation was indeed inadequate because of resource shortages in the whole country.

## Contribution made to the households from the food basket

The monthly ration of the feeding basket consisted of the following (Table 3).

**TABLE 3:** Monthly ration of the feeding basket.

Commodity	Ration (kg)
Cereal	12.5
Pulses	1.8
Vegetable oil	0.6

*Source*: Christian Care, 2010, *Vulnerable group feeding end programme of term report*, Christian Care, Harare

The ration was given out to complement existing household food stocks and not for nutritional measures of the communities. It was established that most of the women (68%) depended on the food basket for themselves and their households. On the other hand, 32% of the respondents indicated that they had other food sources. The food basket lacked diversity as the protein from the pulses constituted low biological value because of the presence of phytate, which inhibits iron absorption into the body (Abbaspour, Hurrell & Kelishadi 2014). Therefore, if pulses were the only source of protein or iron for the respondents they would be prone to malnutrition, leading to diseases like anaemia, one of the main causes of maternal mortality in developing countries (Müller & Krawinkel 2005). Mahan and Escott-Stump (2000) argue that because protein is one of the main components of antibodies, the deficiency of protein could result in a weakened immune system. UNICEF (2007) confirmed that though the food distributed to the beneficiaries was fortified it did not provide adequate micronutrients. The vitamin A that was provided by the vegetable oil was not adequate to meet the nutritional requirements, and this resulted in weak immunity for the respondents and compromised their health. The food basket does not cater for vitamin C, which is also an essential nutrient.

**FIGURE 2:**
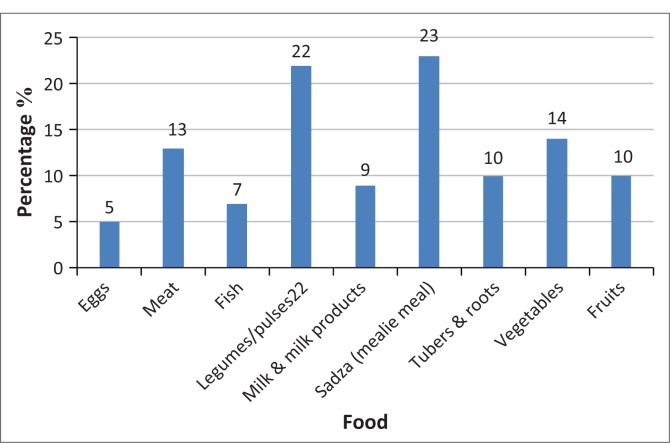
Variety of food eaten on a daily basis.

The respondents were asked to name some foodstuffs that pregnant women usually ate every day in addition to the food basket. This exercise was conducted to investigate whether respondents were following an appropriate diet that could cater for the nutritional requirements during pregnancy. Figure 2 shows how food was consumed on a daily basis. Fish and eggs hardly featured in their diets. The respondents indicated that they consumed eggs (5%), meat (13%), fish (7%), legumes or pulses (22%), milk and milk products (9%), sadza (mealie pap) (23%), tubers and roots (10%), vegetables (14%) and fruits (10%). The above statistics indicated that the respondents relied mainly on the food basket provided because very few had other foodstuffs to eat. Pregnant women require food products for a number of functions such as iron and proteins for a healthy immune system, an increased volume of blood, the growth of the foetus, tissue maintenance and the production of antibodies. The respondents who indicated that they had fish in their diets revealed that the fish was acquired from Mozambique’s Cahora Bassa Dam. This demonstrated some form of supplementary food provision by the respondents, whose spouses and relatives got the fish from the dam.

## Nutritional status

The nutritional status of the women could be determined by anthropometry as it was regarded as a biological outcome of the nutritional security level (Zahid Khan et al. 2013). In order to determine the nutritious welfare of women who are pregnant, an anthropometric indicator should be utilised; these include height, weight and MUAC. Other contributory effects that can disturb the nutritional status of pregnant women were taken into consideration. These included an inadequate diet and the diseases the respondents suffered from 2 weeks prior to the survey. The information was obtained from the nurses in charge of the health institutions. The results of the study revealed that only 32% of the respondents benefited from vitamin A supplementations; however, many of the respondents (68%) were not given supplementation as confirmed by the nurses in charge. According to WHO and WFP standards, less than 22 cm of MUAC was a total indication of malnutrition, and 24% of the women in the study fell in this category.

## Anthropometric findings

The women’s records were obtained from the clinic through the nurses in charge. The findings showed that the respondents’ height was between 1.44 m and 1.76 m. The range of their weight was between 48 kg and 72 kg. Although weight and height could be the result of genetics, the diet of an individual because they were young was another causative element (Rivera 2003). The weight range of the pregnant women revealed that some of them were underweight, with their BMI ranging from 20 to 26.3. The recommended BMI according to WFP (2004) is 19 to 21 for women who are not pregnant and come from developing countries, excluding the Samburu of Kenya, which is 17.6 and the Dinka of South Sudan, which is 17.6. Nevertheless, BMI was hardly applied in anthropometric surveys for pregnant women because it added some extra work in interpreting the extra weight gained.

The results of respondents who had an MUAC of greater than or equal to 22 cm were better (76%) than those with an MUAC of less than 22 cm (24%), which the WFP (2000) indicates as malnutrition. According to the Sphere handbook (2011), an MUAC of smaller than 20.7 cm is an indication of a serious threat of malnourishment. In the study, only two respondents had an MUAC smaller than 20.7. This was out of a total of 24 respondents with an MUAC of less than 22 cm which indicates the dominance of malnutrition among pregnant women in Mbire. The result of the study is in line with statistics of the malnourishment rate of Zimbabwe at 33.8% (Zimbabwe National Nutrition Survey 2010).

## General findings of the study

A lack of variety and the quality of the food that the pregnant women were consuming resulted in failure to achieve the recommended dietary allowances for women who were pregnant. The WFP-VGF food basket complements existing diets and is not the main source of food for the respondents and their households. The VGF feeding basket could not be expected to meet all the nutritional requirements of pregnant women in Mbire.

### Recommendations

#### Household and community level

The partnerships with the Ministry of Agriculture, Mechanisation and Irrigation Development, Agriculture and Extension Services (AGRITEX), the Lower Guruve Development Association (LGDA) and other non-governmental organisations could assist in initiating food safety and nutritional measures such as poultry, orchards, vegetable gardens and goat rearing projects. These projects can assist the vulnerable people, including pregnant women, to gain access to a sufficiently balanced diet.

#### National level

The Ministry of Health in Zimbabwe launched the Expanded Programme on Immunisation (EPI) as a means to intensify micronutrient supplements through health facilities to minimise the dangers associated with a lack of micronutrients, such as impaired immunity and anaemia. It is recommended that such programmes must be intensified to cover areas like Mbire district.

#### World Food Programme level

The WFP could work together with the government through the health ministry to assist with the special programmes targeting pregnant women to reduce complications associated with maternal challenges, thereby reducing maternal and child mortality and morbidity rates. The WFP resources would help the government augment its effort to reduce or even eradicate maternal challenges facing women in Mbire and in Zimbabwe as a whole.

## Conclusion

Although vulnerable pregnant women in Mbire received food aid, these women, especially adolescents and those who had short inter-pregnancy intervals, lacked some micronutrients needed during pregnancy. Their health as well as the health of their unborn babies was compromised. This indicated that the Zimbabwean government was challenged in its effort to achieve Millennium Development Goals (MGDs) 1 (eradication of extreme poverty and hunger) and 5 (improving maternal health). It is yet to be seen what measures the country will implement to attain the Sustainable Development Goals (SDGs).
